# Factors Associated with Management Outcome of Incomplete Abortion in Yirgalem General Hospital, Sidama Zone, Southern Ethiopia

**DOI:** 10.1155/2018/3958681

**Published:** 2018-09-20

**Authors:** Achamyelesh Gebretsadik

**Affiliations:** School of Public Health, College of Medicine and Health Science, Hawassa University, Hawassa, Ethiopia

## Abstract

**Background:**

Each year, several millions of women who underwent abortion also bear several consequences, including infection, massive blood loss, chronic pelvic pain, infertility, and death. Poor treatment outcomes also cause disability and death. The aim of this study was to determine factors associated with management outcomes of incomplete abortion in Yirgalem General Hospital.

**Methods:**

Health facility-based cross-sectional study design was used. Medical record review of 186 women who received abortion service from July 1^st^ 2015 to June 30 2017 was done. Then the data were entered into the computer using epi info version 7.2 and exported into SPSS version 20, Descriptive analysis was done to determine social, demographic characteristics, and bivariate and multivariate logistic regression analysis were done to identify factors associated with management outcome of incomplete abortion, 95% CI and odds ratio used to present the result.

**Results:**

A total of 180 (96.7%) of cases managed for incomplete abortion was included in the study. Of this, 53.3% of patients with incomplete abortion belonged to age group of 18–25 years old. More than half incomplete abortion cases were managed surgically 122 (67.8%). Of the total, 36 (19.4%) of the patients developed unfavorable management outcome. Gestational age at which abortion occurs AOR = 3.39, 95% (1.29, 8.89) and delayed seeking of medical help AOR = 2.96, 95% (1.04, 8.4) were found to be significantly associated with unfavorable management outcome.

**Conclusion:**

High numbers of cases managed for abortion resulted unfavorable management outcome. However, no death occurred and major surgery done as the result of abortion management. Delayed seeking of medical care and seeking care past 1st trimester are significantly associated with unfavorable management outcomes. Therefore, awareness creation for adolescent and youth about prompt health-care seeking after the start of the first sign and symptom of spontaneous abortion should be strengthened.

## 1. Introduction

To address maternal deaths and morbidity from abortion linked complications, in 2005, the Ethiopian Parliament expanded the indications for legal induced abortion to include cases of pregnancy from rape or incest, incurable fetal deformities, women's physical or mental disabilities, preservation of women's life or health, and lack of physical or mental preparedness for childbirth due to young age (<18 years). Prior to the 2005 reforms, pregnancy terminations were permitted to avoid physical comorbidities and mental disease [1].

In Ethiopia, the proportion of abortion-related service provided by midlevel health workers increased nearly double the proportion in 2008 (27%) and 53% in 2014. In 2014, almost three-fourths of facilities that could potentially provide abortions or postabortion care did so, including 67% of the 2,600 public health centers nationwide, 80% of the 1,300 private or nongovernmental organization (NGO) facilities, and 98% of the 120 public hospitals provide abortion and postabortion care in Ethiopia [2].

Since 2009, the total number of women sought abortion care in public health facilities is increasing. Two-thirds of the women received safe and legally induced abortion care and the remaining received postabortion care for complication of unsafe abortion [2].

Treatment failures of abortion can occur due to incompleteness of expulsion of products that cause excessive bleeding, infection due to ascending route of microbial migration, perforation of genital organ, also causing blood loss, and infection in addition to long-term problems like infertility [3]. The morbidity and all consequences of abortion have an impact on the life of individual, family, society, and country as a whole. The consequences of abortion costs several budget, long-term disabilities like prolonged hospital stay and sterility [4]. A study showed the efficacy of management methods of abortion vary based on the types of treatments. Success of treatment was higher in surgical methods than medical one [5]. In contrast, a number of studies conducted explaining that surgical techniques were more risky than medical intervention methods [6–12].

Many researches regarding abortion in Ethiopia focus on the magnitude and types of abortion, whereas studies showing management outcomes of incomplete abortion and factors associated with it are very limited. Therefore, this study was done to assess factors associated with management outcome of incomplete abortion in Yirgalem General Hospital, and the result helps to improve the management of incomplete abortion.

## 2. Methods

### 2.1. Study Area

The study was conducted in Yirgalem general hospital, which is found in a Sidama zone in Southern Nation Nationalities People Region Ethiopia. It is located 322 km south of the capital Addis Ababa and 47 km southeast of regional capital Hawassa. It serves for a catchment population of 4.2 million, mainly from Sidama zone and other surrounding areas. It serves about 65,222 patients of all types every year. Obstetrics and gynecology is one of the major departments of the hospital where different services such as delivery, abortion care, different gynecological surgical care, and family planning are offered. In this hospital, first trimester abortion management is done using surgical methods like (manual vacuum aspiration, dilatation and curettage, and evacuation and curettage) and medical methods like vaginal and oral misoprostol drug. In addition, for management of complicated cases, laparotomy and hysterectomy are also done.

### 2.2. Study Design and Period

A health facility-based cross-sectional study was conducted. The study was conducted from July 1^st^ 2015 to June 30 2017 at Yirgalem General Hospital.

### 2.3. Source and Study Population

Source population was all those who presented to the hospital with incomplete abortions with no outside interference cases from July 1^st^ 2015 to June 30^th^ 2017 and had received care in Yirgalem General Hospital.

### 2.4. Inclusion Criteria

Medical records of women who had a history of pregnancy and clinically appear with vaginal bleeding, including passage of conceptus tissue, but not completely, were included and those who had a history of clinical future which indicates a complication at the time of first presentation were excluded from the study.

### 2.5. Sample Size Determination

The sample size of the first objective is calculated based on the following assumption.

- Level of confidence of 95%, thus *Z* (1−*α*/2) = 1.96
- Margin of error of 5%, thus *d*=0.05

*P*=11.9% is the commonest complication of abortion (*p*) for outcome of abortion as obtained by a study done in Jimma university teaching hospital [13].

Then sample size “*n*” was calculated with epi info version 7, *n*=162. Adding 10% contingency taking account for missing data and incompleteness of charts gives the final sample size of *n*=178.

The sample size for the second objective is calculated by taking estimated proportions of the most common risk factors determining management outcome of incomplete abortion (duration of vaginal bleeding <3 days = 80% and OR = 7.5) according to a study conducted in Adigrat Zonal Hospital, Tigray Region, Northern Ethiopia [14].

By using epi info statistical software version 7, sample size of the second objective is calculated with epi info version 7, *n*=104. Adding 10% contingency taking account for missing data and the incompleteness of charts gives the final sample size of *n*=114.

Therefore, the calculated sample sizes for first & second objectives are 178 and 114, respectively. However, the overall cases managed for incomplete abortion within that specified period in the hospital were 186 cases; as a result, all abortion cases included in the study.

### 2.6. Sampling Procedure/Technique

All records with the diagnosis of incomplete abortion in gynecology inpatient, outpatient and delivery ward registration books were traced by reviewing and collecting the card number & names of all cases in the proposed study period. In the proposed study period, 186 abortion cases have been seen in Yirgalem general hospital. Among these, 4 cards were avoided because of incompleteness and 2 cards were missing, so that data were collected from 180 patient records and all necessary data were reviewed using checklists.

### 2.7. Study Variables

#### 2.7.1. Dependent Variables

Management outcome of incomplete abortion (favorable/unfavorable) was considered the dependent variable.

#### 2.7.2. Independent Variables

- Age
- Place of residence
- Gravidity, gestational age, and previous history
- Contraceptive use, marital status, parity, and duration of the problem
- Method of initial management and time of seeking care after the onset of the symptoms.

### 2.8. Method of Data Collection

The patient's medical record numbers from obstetrics and gynecologic outpatient department, inpatient department, and operation theatre registration book were reviewed to trace patients treated for incomplete abortion cases from July 1^st^ 2015 to June 30^th^ 2017. The records were thoroughly reviewed for signs and symptoms of incomplete abortion during the time of hospital presentation, after initial management from progress and discharge report. Four health workers working in obstetrics and gynecology ward collected the data after adequate training was given, with a semistructured checklist. Supervision was undertaken by the investigator.

#### 2.8.1. Operational Definitions

*Outcome*. Condition of patients at discharge (both improved and no postabortion complication or improved, but developed one or more complications after the initiation of the initial management or dead).

*Favorable Outcome*. Patients with a clinical diagnosis of incomplete abortion improved and discharged from the hospital and developed no complication.

*Unfavorable Outcome*. Patients with a clinical diagnosis of incomplete abortion whose condition is improved, but developed one or more complication after the initiation of the initial management(s), e.g., shock, sepsis, uterine, bowel and bladder perforation, laparotomy, hysterectomy, or patients with moderate or severe complication or with a clinical diagnosis of incomplete abortion who have died in the hospital.

- Categories of severity of complication
    No complication: patients who show no sign of complication
     (a) Normal body temperature (35.5–37.5) degree Celsius
     (b) No clinical sign of infection plus
     (c) No systemic or organ failure plus
     (d) No suspicious finding on evacuation
   
*Moderate:* patients who show one or more of the following complications
     (a) Body temperature 37.6–37.9 degree Celsius
     (b) Offensive vaginal discharge
     (c) Localized peritonitis
     (d) Pulse rate 100–120/min
   
*Severe*: patients who having one or more of the following complications
     (a) Body temperature > 38 degree celsius
     (b) Organ failure
     (c) Generalized peritonitis
     (d) Pulse rate > 120 beat/min
     (e) Foreign body/mechanical injury on evacuation
     (f) Death

For identification and categorization of sign and symptoms, documents were reviewed. Consequently, those who had presented at least one sign or symptom were categorized based on the respected classification.

### 2.9. Method of Data Analysis

Data were fitted for their completeness and recorded using the SPSS-20 database program for analysis after edition. Descriptive analysis was performed along the sociodemographic, symptoms, signs, and consequences of abortion. Bivariate analysis was applied to all variables. And a multivariable logistic analysis was also done for variables which showed a significance level of 0.2 or below in the bivariate analysis to see the relationship between the dependent and independent variables. 95% confidence interval and odds ratio were applied to quantify risk of having unfavorable outcome of abortion. Hosmer–Lemeshow goodness of test was used to fit the model. All the independent variables also checked for the existence of any interaction. Sociodemographic and clinical sign and symptoms and methods of management used variables were included in the models.

### 2.10. Data Quality Assurances

To maintain the quality of information, data collection was formed by four experienced health workers and detail training for one day was given to all. During data collection, day to day activities were supervised and evaluated for errors to be corrected by the investigator. The checklist was retested on Hawassa referral hospital, and after the tests, the checklist was modified.

## 3. Results

The majority of patients were rural residents (72.8%), and the mean age is 24.69 with (SD ± 5.79). Most of them, 96 (53.3%), were found in the age group of 18–25 years. In conditions of marital status majority, 132 (73.3%), were married and the rest are single. Among all medical records reviewed, more than half of women were multigravida, 101 (56.1%), and multiparous as well 77 (42.8%). Among all reviewed, 60 (33.3%) were significant for the foremost time (Table 1).

The majority of patients were presented with vaginal bleeding 142 (78.9%) and presented to the facility within the first 24–72 hrs after bleeding 66 (36.7%). Ninety-five (52.8%) of them were under less than 12 weeks of gestation. Thirty-two percent (58) were managed by medical methods using misoprostol drug and 122 (67.8%) were managed by surgical methods. Of those, 108 (88.5 %) of all cases was managed by manual vacuum aspiration (MVA), whereas 14 (11.5%) were managed by sharp metallic curettage (E&C). Of the pregnancies under 12 weeks of gestation, 71 (74.7%) had uterine evacuation using manual vacuum aspiration and 15 (15.8%) had managed medically (Table 2).

### 3.1. Complications and Management Outcomes of Incomplete Abortion

Thirty-six (19.4%) patients had been made out for severe complications of miscarriage. In this regard, 22 (62.8%) patients received cross-matched blood. Among these transfused patients, 20 of them were in hypovolemic shock and severe anemia, and the remaining two patients were transfused for an indication of sepsis secondary to septic abortion. The other 6 (17%) patients who developed severe complication were diagnosed with hypovolemic shock secondary to blood loss but treated without blood transfusion. The remaining 7 (20%) patients among those developed severe complications were diagnosed with septic abortion but not transfused with blood.

**Figure 1:**
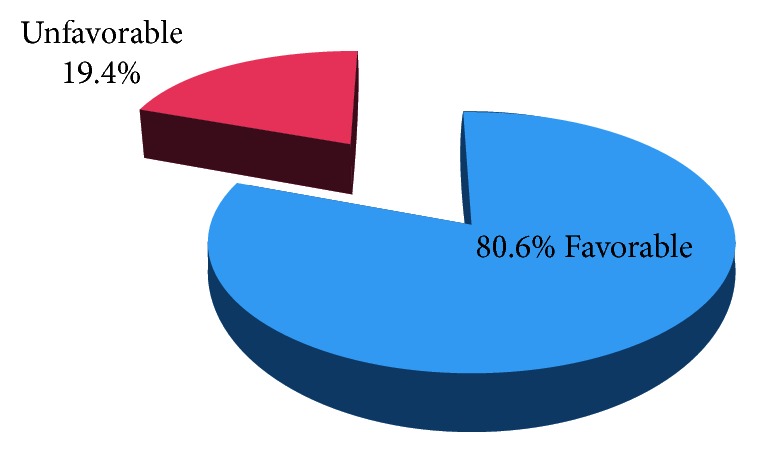
Management outcomes of incomplete abortion at Yirgalem General Hospital, Sidama zone, southern Ethiopia, from 1^st^ July 2015 to July 30^th^ 2017.

From all incomplete abortion cases evaluated in the hospital that did not end up with death, laparotomy, or hysterectomy, 3 patients were referred to nearest referral hospital for indication of multiorgan failure following septic shock and lack of blood to transfuse (Figure 1).

### 3.2. Factors Determining Management Outcomes of Incomplete Abortion

Demographic, reproductive, and other factors were investigated using bivariate and multivariate analysis. Among the listed characteristics, gestational age of the pregnancy and duration of stay before medical attention were found to be associated when investigated with bivariate and multivariate regression. Accordingly, gestational age of more than 12 weeks showed association AOR about 3.39, 95% CI (1.29, 8.89). In this case, women who were after 12 weeks of gestation age are more than 3 times higher to develop unfavorable outcome for incomplete abortion management. This study also showed that women who stayed for more than 72 hours before the medical visit after they developed clinical manifestation of abortion are at risk of developing unfavorable outcome of abortion with AOR of 3.08,95% CI (1.08, 8.78).

Similarly, surgical method of initial management is found to be associated AOR with 2.96, 95% CI (1.04, 8.40) (Table 3).

## 4. Discussion

In this study, 19.4% of cases developed unfavorable outcome. In general, there was no death or major surgery found in this study. The study also found that gestational age beyond 12 weeks and staying for more than 72 hours before the hospital visit were found to have the risk of having unfavorable management outcomes of incomplete abortion.

This study showed 19.4% of incomplete abortion patients developed unfavorable outcome. This result is in agreement with study done in Malawi [15] and lower than a study done in Tikur anbessa specialized hospital. This might be explained by the difference in the level of hospitals [9]. This national load of referred cases and presence of several complicated cases may explain the death recorded in Tikur anbessa hospital. Concerning the complications of incomplete abortion management, this research finding agrees with research update of maternal mortality trends in Ethiopia which showed decreased rate of death due to abortion as there was no death recorded due to incomplete abortion in Yirgalem General Hospital in the study period [2]. However, the proportion of unfavorable outcome is still high.

Incomplete abortion occurring in gestational age beyond 12 weeks had 3.4 times more risk developing unfavorable management outcome. This finding is in line with several studies' findings conducted in Ethiopia [9, 10, 14]. This is because as the fetus in the uterus grows older, the size of conceptus and blood supply is increased very much which in turn results in extensive bleeding and uterine manipulation, which harms the mother and make outcome of abortion management unfavorable. World Health Organizational research also agrees by stating the older the gestational age, the worst will be the outcome of abortion treatment. This study showed more number of maternal death and severe complications that required major surgeries in women who aborted after gestational age of 14 weeks [7]. Similarly, the Indian study also showed that incomplete abortion occurred after 12 weeks resulted in loss of life of mother, morbid surgery, and irreversible sequel because of it [8].

Delay to get medical attention or treatment is associated with unfavorable management outcome of incomplete abortion. Numerous studies done in local and outside like India, Ghana, Kenya, and Malawi are also in agreement with this result [3, 8, 9, 15, 16], which can be explained by the fact that the longer a patient stays after the occurrence of symptoms, the worst will outcome of the treatment for abortion.

### 4.1. Limitation of the Study

This study is based on secondary data of patient medical records which lack several important information like educational level, level of income, and occupation. In addition to that, all the signs and symptoms of the patients might not be recorded by the physician or any concealed sign and symptoms might not be reported by the patients; thus, these might lead to inaccurate classification of outcome variable. Important variables like types of initial management were insignificant in our study, which is likely because of our sample size was relatively small.

## 5. Conclusion and Recommendations

This study found that the magnitude of unfavorable outcome of incomplete abortion treatment is high as compared to studies done in other parts of the country.

Regarding the factors determining unfavorable management outcome of incomplete abortion, gestational age of more than 12 weeks and delay of patients to get medical help are found to be associated. Therefore, raising awareness of women in reproductive age group to avoid delay seeking of abortion care after the start of the first sign and symptom of spontaneous abortion should be strengthened.

## Figures and Tables

**Table 1:** Demographic and reproductive characteristics of women who received care for incomplete abortion in Yirgalem General Hospital, southern Ethiopia, from July 1^st^ 2015 to June 30^th^ 2017 (*N*=180).

Characteristics	Frequency	Percentage
Residence		
Urban	49	27.2
Rural	131	72.8

Age		
Less than 18 years old	22	12.8
From 18 to 25 years	96	53.3
From 26 to 35 years	54	30
Above 35 years	8	4.4

Marital status		
Single	48	26.7
Married	132	73.3

Gravidity (number of gestation)		
Primi gravida	60	33.3
Multi gravida	101	56.1
Grand multi	**19**	10.6

Parity (number of deliveries)		
Nuli para	69	38.3
Primi para	29	16.1
Multi para	77	42.8
Grand multi	5	2.8

**Table 2:** Clinical presentation and methods of management of incomplete abortion in Yirgalem General Hospital, southern Ethiopia, from July 1^st^ 2015 to June 30^th^ 2017 (*N*=180).

Factors	Frequency	Percentage
Gestational age		
<12 weeks	95	52.8
>12 weeks	85	47.2

Clinical presentation		
Vaginal bleeding	142	78.9
Others (abdominal pain)	38	21.1

The duration of the problem		
<24 hours	75	41.7
24–72 hours	66	36.7
More than 72 hours	39	21.7

Method of initial management		
Medical	58	32.2
Surgical	122	67.8

Types of surgical management		
MVA	108	88.5
E&C	14	11.5

Blood transfusion		
Yes	22	12.2
No	158	87.8

Length of hospitalization		
<1 day	84	46.7
2-3 days	73	40.6
4-5 days	23	12.8

**Table 3:** Multivariate analysis result of factors affecting management outcomes of incomplete abortion among in Yirgalem General Hospital, southern Ethiopia, from July 1st, 2015 to July 30^th^ 2017 (*n*=180).

Factors	Management outcome			
	Favorable count (%)	Unfavorable count (%)	COR (95% CI)	AOR (95% CI)
Age of the women				
<18	20 (90.9%)	2 (9.1%)	1	1
18–25	80 (83.3%)	16 (16.7%)	2 (0.42, 9.41)	1.58 (0.28, 8.82)
26–35	37 (68.5%)	17 (31.5%)	4.59 (0.96, 21.92)	3.38 (0.51, 22.09)
>35	7 (87.7%)	1 (12.5%)	1.42 (0.11, 18.29)	0.55 (0.28, 10.78)

Residence				
Urban	43 (87.8%)	6 (12.2%)	1	1
Rural	101 (77.1%)	30 (22.9%)	2.12 (0.82, 5.48)	1.3 (0.45, 3.77)

Gravidity				
Primi gravid	52 (86.7)	8 (13.3%)	1	1
Multi gravida	78 (77.2%)	23 (22.8%)	1.91 (0.79, 4.6)	1.57 (0.54, 4.55)
Grand multi gravida	14 (73.7%)	5 (26.3%)	2.32 (0.65, 8.21)	0.88 (0.16, 4.85)

Gestational age				
<=12 weeks	83 (87.4%)	12 (12.6%)	1	1
>12 weeks	61 (71.8%)	24 (28.2%)	**2.72 (1.26, 5.86)** ^*∗*^	**3.39 (1.29, 8.89)** ^*∗*^

Time of seeking care after the onset of the symptoms				
<24 hours	63 (84%)	12 (16%)		1
24–72 hours	57 (86.4%)	9 (13.6%)	0.97 (0.32, 2.11)	0.94 (0.33, 2.65)
>72 hours	24 (61.5%)	15 (38.5%)	**3.28 (1.34, 8.01)** ^*∗*^	**3.08 (1.08, 8.78)** ^*∗*^

Methods of initial management				
Medical	46 (79.3%)	12 (20.7%)	1	1
Surgical	98 (80.3%)	24 (19.7%)	0.94 (0.43, 2.04)	0.94 (0.35, 2.5)
